# How to involve carers in the acute care: An online training for clinicians across four sites in England

**DOI:** 10.1192/j.eurpsy.2022.1562

**Published:** 2022-09-01

**Authors:** E. Petkari, A. Kouroupa, D. Giacco

**Affiliations:** 1 Universidad Internacional de La Rioja, Health, Logroño, Spain; 2 University College London, Psychiatry, London, United Kingdom; 3 University of Warwick, Wms - Health Sciences, Warwick, United Kingdom

**Keywords:** acute service, online training, severe mental illness, carer involvement

## Abstract

**Introduction:**

Involving carers in the care of people with severe mental illness is known to bring positive treatment and psychosocial outcomes. However, evidence-based procedures to guide clinicians on how to involve carers in the acute care are lacking.

**Objectives:**

To provide an online training to clinicians working in the acute care regarding the organisation of a standardised meeting with the service user and their carer within the first week of hospitalisation, and explore their views after its implementation.

**Methods:**

We trained six clinicians across four urban and rural sites in England, asked them to incorporate the meeting in their routine care provision and interviewed them to explore their experiences.

**Results:**

Clinicians reported training advantages such as ease of use, comprehensiveness and transferable skills, and meeting advantages such as shared goals development and acknowledgement of carer involvement value. They also mentioned challenges related to organisational/time constraints, expectations management, and distance to the hospital for carers. Clinicians suggested to further focus on carer motivation to engage, to use skills throughout admission rather than in a one-off session, and to provide a structured meeting summary. Those experiences were shared across sites, indicating similar benefits and challenges, not depending on the specific setting characteristics.

**Conclusions:**

Providing structured training to clinicians may increase carer involvement in routine care in acute settings. Given the workload in such settings training endeavours should be brief and include skills that clinicians can apply to facilitate shared goal development and expectations management. The use of online meetings may allow increased carer participation in the acute care.

**Disclosure:**

No significant relationships.

